# Socio-Demographic Characteristics and Body Weight Perceptions of Study Participants Benefitting Most from the Feel4Diabetes Program Based on Their Anthropometric and Glycaemic Profile Changes

**DOI:** 10.3390/nu12103117

**Published:** 2020-10-13

**Authors:** George Moschonis, Kalliopi Karatzi, Kyriaki Apergi, Stavros Liatis, Jemina Kivelä, Katja Wikström, Alelí M. Ayala-Marín, Rocio Mateo-Gallego, Kaloyan Tsochev, Nevena Chakarova, Emese Antal, Imre Rurik, Violeta Iotova, Greet Cardon, Jaana Lindstrom, Luis A. Moreno, Konstantinos Makrilakis, Yannis Manios

**Affiliations:** 1 Department of Dietetics, Nutrition and Sport, School of Allied Health, Human Services and Sport, La Trobe University, Melbourne, VIC 3086, Australia; g.moschonis@latrobe.edu.au; 2 Department of Nutrition and Dietetics, School of Health Science and Education, Harokopio University, 17671 Athens, Greece; pkaratzi@hua.gr (K.K.); kiapergi@gmail.com (K.A.); 3 National and Kapodistrian University of Athens Medical School, 11527 Athens, Greece; s.liatis@yahoo.com (S.L.); kmakrila@med.uoa.gr (K.M.); 4 Department of Public Health Solutions, Finnish Institute for Health and Welfare, 00271 Helsinki, Finland; jemina.kivela@thl.fi (J.K.); katja.wikstrom@thl.fi (K.W.); jaana.lindstrom@thl.fi (J.L.); 5 Growth, Exercise, Nutrition and Development (GENUD) Research Group, Instituto Agroalimentario de Aragón (IA2), Instituto de Investigación Sanitaria de Aragón (IIS Aragón), Universidad de Zaragoza, 50009 Zaragoza, Spain; 751405@unizar.es (A.M.A.-M.); lmoreno@unizar.es (L.A.M.); 6 Centro de Investigación Biomédica en Red Enfermedades Cardiovasculares (CIBERCV), Instituto de Salud Carlos III, 28029 Madrid, Spain; rmateo@unizar.es; 7 Department of Paediatrics, Medical University Varna, 9002 Varna, Bulgaria; kalooyan@abv.bg (K.T.); violeta.iotova@mu-varna.bg (V.I.); 8 Clinical Center of Endocrinology, Department of Diabetology, Medical University Sofia, 1431 Sofia, Bulgaria; veni_chakarova@abv.bg; 9 Hungarian Society of Nutrition, 1088 Budapest, Hungary; diet.Emese.Antal@gmail.com (E.A.); rurik.imre@med.unideb.hu (I.R.); 10 Department of Movement and Sports Sciences, Faculty of Medicine and Health Sciences, Ghent University, 9000 Gent, Belgium; Greet.Cardon@UGent.be

**Keywords:** Feel4Diabetes, lifestyle intervention, prevention, type 2 diabetes, obesity, glycaemic indices, glucose, community, school, families

## Abstract

The Feel4Diabetes program was comprised of a community-based screening and a two-year intervention phase aiming to prevent type 2 diabetes (T2D) in families at risk for diabetes across Europe. The current work aimed to identify the socio-demographic characteristics and body weight perceptions of participants who benefitted the most, achieving at least a 5% reduction in body weight, waist circumference and glycaemic indices (fasting plasma glucose, insulin, glycosylated haemoglobin levels), over two-year period. Following a two-stage screening procedure, 2294 high-risk parents were randomly allocated to standard care or more intensive intervention. The participants who benefitted most were living in Southern (OR 2.39–3.67, *p* < 0.001) and Eastern Europe (OR 1.55–2.47, *p* < 0.05), received more intensive intervention (OR 1.53–1.90, *p* = 0.002) and were younger (<40 years old) adults (OR 1.48–1.51, *p* < 0.05). Furthermore, individuals with tertiary education (OR 2.06, *p* < 0.001), who were unemployed (OR 1.62–1.68, *p* < 0.05) and perceived their body weight to be higher than normal (OR 1.58–3.00, *p* < 0.05) were more likely to benefit from the program. Lastly, males were more likely to show improvements in their glycaemic profiles compared to females (OR 1.40, *p* = 0.024). These findings point out the regions in Europe and the sociodemographic profile of individuals that benefitted the most in the current study, highlighting the need to prioritise regions in greater need for such interventions and also tailor future interventions to the characteristics and perceptions of the target populations.

## 1. Introduction

Ten percent of the global population over 25 years of age suffers from type 2 diabetes (T2D) [1]. According to the International Diabetes Federation, it is projected that by 2030 more than 470 million people worldwide will be affected by prediabetes [2] and that by the year 2045 there will be about 700 million T2D patients [3]. Although clinical trials have demonstrated the effectiveness of glycaemic control in reducing or delaying the long-term complications of T2D [4], the public health burden of the disease is still rising, posing direct and indirect financial overload on healthcare systems and on society overall. For these reasons, there is a need for successful and cost-effective T2D preventive program strategies.

There is a disproportionally higher prevalence of T2D and its risk factors among certain population groups [5,6,7,8,9,10]. In this regard, a large segment of the population in low- and middle-income countries, but also certain groups with low socio-economic status (SES) in high income countries, such as minority groups and immigrants, have been reported to be at a higher risk for developing T2D [11]. Low SES has been associated with a higher prevalence of T2D, overweight/obesity and unhealthy energy balance-related behaviours (EBRBs), such as physical inactivity, sedentary behaviour and unhealthy dietary habits [5,6,7,8,9,10]. As EBRBs represent modifiable risk factors for T2D, any program aiming to prevent the occurrence of the disorder should primarily focus on groups with characteristics and behaviours that are linked to low SES.

However, the effectiveness of lifestyle programs in the prevention of T2D is varying [12,13,14,15,16,17]. This is partly due to the variability in the frequency and intensity of the implemented programs and partly due to their implementation in the wider population instead of groups at high risk, and as such, most in need of these programs [12,18,19]. This highlights the need for screening the population in order to identify those that would benefit most from lifestyle intervention programs. Understanding the characteristics of individuals who would benefit most from the intervention is an important preliminary step for tailoring the intervention to the needs of its recipients, thus improving its effectiveness in the prevention of T2D.

Recognizing the need for prioritising population groups at risk for diabetes and early screening for identifying high risk families, the Feel4Diabetes program was designed and implemented in six EU member states. The Feel4Diabetes program was comprised of a community-based screening phase and an intervention phase providing either standard care or a more intensive intervention component aiming to promote healthy eating and an active lifestyle in high risk families. The aim of the current work is to identify the socio-demographic characteristics and body weight perceptions of participating parents who benefitted most from the Feel4Diabetes program based on changes in their anthropometric and glycaemic profile indices.

## 2. Materials and Methods

### 2.1. Study Design and Sampling Procedures

The Feel4Diabetes program (http://feel4diabetes-study.eu/, NCT02393872) was a large school- and community-based study aiming to promote a healthy lifestyle including healthy eating and increased physical activity in order to alleviate the negative outcomes of obesity and obesity-related metabolic risk factors in families at risk for T2D in Europe. The actual implementation of the Feel4Diabetes program had a total duration of two years (2016–2018). 

The study was conducted within selected provinces in six European countries and the recruitment was based on a standardised, multi-stage sampling procedure, targeting population groups at high risk for developing T2D. Specifically, in Bulgaria (i.e., Low and Middle Income Country: LMIC), all the municipalities within the participating regions were eligible for recruitment, while in Belgium, Finland, Greece and Spain (i.e., High Income Countries: HICs), families within low SES municipalities were recruited. Specifically, in HICs, low SES municipalities were defined as those with the lowest educational level and/or the highest unemployment rates, as retrieved from official resources and local authorities within each country. More details regarding study design can be found elsewhere [20].

In brief, primary schools located in the selected municipalities were used as the entry-point to the community and all parents having children in the first three grades of primary school were invited to go through a self-reported screening procedure with the use of the FINDRISC questionnaire [21]. Families with at least one “high-risk parent” (having a high FINDRISC score) were defined as “high-risk families” and the adult members of these families were invited to go through a brief medical check-up in local community centres. Depending on the municipality these families were living in (intervention or control municipality), they further received the standard care or a more intensive intervention. Standard care was comprised of a brief medical check-up and one counselling session for the adult members of the families annually. Intensive intervention was comprised (beyond the annual medical check-ups) of six counselling sessions the first year and a motivational feedback and guidance via SMS intervention the second year. Furthermore, a school-based intervention was delivered for all families in the intervention municipalities. A detailed description of the study design and the screening procedures followed can be found in relevant previous published articles from the Feel4Diabetes study [20,22].

### 2.2. Ethical Approvals and Consent Forms

The Feel4Diabetes study adhered to the Declaration of Helsinki and the conventions of the Council of Europe on human rights and biomedicine. Prior to initiating the program, all participating countries obtained ethical clearance from the relevant ethical committees and local authorities. All participants read a detailed description of the study and filled in consent forms for their participation but had the chance to withdraw from the study at any point. More details regarding the approvals received by each country can be found elsewhere [23].

### 2.3. Measurements

In order to assess the socio-demographic characteristics and body weight perceptions of study participants, data were collected at baseline (2016), first (2017) and second year (2018) of the program by well-trained researchers [24].

#### 2.3.1. Socio-Demographic Characteristics and Body Weight Perceptions

Information on demographic and socio-economic characteristics (e.g., age, gender, paternal and maternal years of education, race, marital and employment status) of the families participating in the Feel4Diabetes program was collected using self-reported questionnaires in all study participants. All continuous variables related to participants’ socio-demographics were dichotomized as follows: age: <40 years vs. ≥40 years, education: >12 years vs. ≤12 years (12 years is the threshold between secondary and tertiary education).

The variable that assessed parents’ weight perception was dichotomized into a new variable as follows: my body weight is higher than normal vs. my body weight is normal or lower than normal.

#### 2.3.2. Anthropometry

Standing height was measured without shoes and was recorded to the nearest tenth of a centimetre (i.e., 0.1 cm) using telescopic stadiometers: SECA 213, SECA 214, SECA 217 and SECA 225. Body weight was measured with light clothing and without shoes and recorded to the nearest 0.1 kg. The equipment included electronic weight scales: SECA 813 and SECA 877. Body mass index (BMI) was calculated by dividing body weight (kg) to height squared (m^2^). Waist circumference (WC) measurements were recorded to the nearest tenth of centimetre (i.e., 0.1 cm) using a non-elastic measuring tape (SECA 201).

#### 2.3.3. Glycaemic Profile Indices

Blood samples were collected by qualified phlebotomists through venipuncture in the morning after an overnight fasting period of at least 8 h. Measurements of fasting plasma glucose (FPG), fasting serum insulin and glycosylated haemoglobin (HbA1c %) were obtained. Blood samples directed for glucose measurement were collected in tubes with sodium fluoride (10.0 mg) and potassium oxalate (8.0 mg) for the inhibition of glycolysis. For the insulin measurements, blood samples were collected in tubes that contained a clotting activator and gel for serum separation. For Hba1c measurements, blood samples were collected in tubes with added anticoagulant (i.e., K3 EDTA). Blood samples directed for serum separation were kept in an upright position for a waiting period ranging between 30 and 120 min at room temperature (20–22 °C), where blood was left to clot before centrifugation. Clotted and non-clotted blood samples were centrifuged at 2000–2200 g RCF (relative centrifugal force) for 10 min for serum and plasma separation, respectively. The whole blood samples that were directed for HbA1c measurement were placed in transportation boxes in an upright position and were transferred to the collaborating accredited laboratories in each study centre. The serum or whole blood samples that were about to be analysed within 8 h were stored at room temperature (20–22 °C), those to be analysed within 4 weeks were stored at −20 °C, while samples that were to be analysed later than 4 weeks were stored at −80 °C. In all the study centres, biochemical analyses were conducted in accredited laboratories following the same standardised procedures and using the same assay kits. Plasma glucose was measured by standard enzymatic procedures, insulin was measured by Electrochemiluminescence (ECLIA) method and HbA1c was determined via high-performance liquid chromatography. For all three glycaemic profile markers the intra-assay coefficients of variation were within the acceptable thresholds in all study centres (i.e., <2.2% for FPG; <5% for serum insulin; and <5% for HbA1c) [25].

#### 2.3.4. Statistical Analysis

All statistical analyses were performed using the Statistical Package for Social Sciences (SPSS Inc., Chicago, IL, USA), version 25.0. The normality of the distribution of continuous variables was tested by the Kolmogorov–Smirnov test. Normally distributed continuous variables are presented as means and standard deviations (SD), while categorical variables are presented as percentages (%).

Participants benefitting from participating either in the more intensive intervention or in the standard care treatment were defined as those who had ≥5% reduction in body weight, WC, glucose, insulin or HbA1c compared to the corresponding baseline value. The cut-off point of 5% was based on literature showing that even a minimum reduction of >5% in body weight, WC, glucose and HBA1c is identified as clinically significant [26,27,28,29].

Between-group differences of continuous variables were tested using either one-way Analysis of Variance (ANOVA) or the independent samples T-test. The significance of the association between categorical variables was examined using the chi-square test. Finally, multivariate logistic regression analyses were carried out to examine associations between benefitting from the program (independent variable) and participants’ socio-demographic characteristics and psychosocial factors at baseline (dependent variables). All logistic regression models were adjusted for age, gender, country and treatment arm (except when used as the dependent variable in the analyses). All reported P-values were two-tailed, and the level of statistical significance was set at *p* < 0.05.

## 3. Results

### 3.1. Baseline Characteristics of Study Participants

Table 1 presents the descriptive characteristics of the study participants in terms of their socio-demographics, anthropometrics and glycaemic profile indices in the total sample (*n* = 2294) and by treatment arm. The majority of study participants were female (65.6%), living in southern European countries (42.5%), had a tertiary education of more than 12 years (73.2%), were married (91.7%), Caucasian (94.2%) and employed (78.2%). The study participants had a mean age of 42.9 ± 7.6 years, a mean body weight of 80.5 ± 18.5 kg, a mean BMI of 28.5 ± 5.7 kg/m^2^ and a mean WC of 94.4 ± 7.6 cm. Regarding the glycaemic profile of study participants, their mean FPG was 5.3 ± 1.1 mmol/L, their mean serum insulin was 9.3 ± 8.8 mU/L and their mean HbA1c was 5.5 ± 0.6%. There were no significant differences between treatment arms in any of these characteristics in almost all these baseline characteristics, thus indicating homogeneity at baseline. The only exception was the region of residence, since the percentage of study participants living in southern Europe was higher in those that received the more intensive intervention compared to those that received standard care (42.7% vs. 42.3%, *p* < 0.05). The opposite was observed in northern Europe, where a higher percentage of study participants received the standard care compared to those that received the more intensive intervention (28.9% vs. 25.1%, *p* < 0.05). 

### 3.2. Mean Weight Loss and Percentages of Study Participants Benefitting from the Feel4Diabetes Program

The mean anthropometric values of BMI and WC at baseline, the mean weight loss and the percentages of study participants in the total sample and by country that benefitted from the Feel4Diabetes program (i.e., more intensive intervention or standard care) are summarised in Table 2. At the baseline, study participants from Finland, Spain and Greece were found to have higher mean BMI and WC values (*p* < 0.05), compared to participants from Belgium and Bulgaria. At the end of the first and second year of the program, the country with the highest percentages of study participants benefitting most, based on reductions in their body weight, was Bulgaria (16.6% and 22.2%, respectively). Mean weight loss in those participants that benefitted from the intervention ranged from 7.5 (3.8) Kg in Belgium to 8.6 (6.1) Kg in Spain at the end of the first year of the program and from 7.7 (3.5) Kg in Bulgaria to 10.7 (9.6) Kg in Spain at the end of the second year of the program. In addition, the country with the highest percentages of study participants that benefitted the most, based on reductions in their WC at the end of the first and second year of the program, was Spain (35.8% and 41.1%, respectively). At the end of the first year of the program, study participants in Bulgaria also benefitted the most based on reductions observed in their FPG (25.8%), serum insulin (23.9%) and HbA1c (25.9%). At the end of the second year of the program, study participants in Spain benefitted the most based on reductions in their FPG (25.0%) and HbA1c (22.1%), while study participants in Belgium benefitted the most based on reductions in their serum insulin (19.1%). 

### 3.3. Associations between Sociodemographic Characteristics and Body Weight Perceptions of Study Participants and Benefit Based on Changes in Anthropometric Indices

Table 3 presents the associations between the sociodemographic characteristics and body weight perceptions of study participants and the relevant benefit from the Feel4Diabetes program based on the reductions observed in their body weight or WC. The odds of study participants benefitting most based on the reductions observed in their body weight, were higher for participants that were younger (<40 years of age) (OR 1.49, *p* = 0.011), unemployed (OR 1.62, *p* = 0.014, only in the first year of the program), those living in Eastern Europe (OR 1.56, *p* = 0.016, only in the first year of the program) and those that perceived their body weight as higher than normal (OR 3.00, *p* < 0.001). Furthermore, the odds of study participants benefitting most based on the reductions observed in their WC, were higher for study participants that received the more intensive intervention (OR 1.53, *p* = 0.002), that were living in southern Europe (OR 2.39, *p* < 0.001), had a tertiary education (i.e., >12 years) (OR 2.06, *p* < 0.001), consumed breakfast daily (OR 2.05, *p* < 0.001) and those that perceived their body weight as higher than normal (OR 1.58, *p* = 0.010). On the contrary, the odds were lower for those living in Eastern Europe (OR 0.13, *p* < 0.001).

### 3.4. Associations between Sociodemographic Characteristics and Body Weight Perceptions of Study Participants and Benefit Based on Changes in Their Glycaemic Profile Indices

The associations between the sociodemographic characteristics and body weight perceptions of study participants and the relevant benefit from the Feel4Diabetes program based on the reductions in their FPG, insulin or HbA1c are summarised in Table 4. The odds of study participants benefitting most based on the reductions observed in their FPG, were higher for males (OR 1.40, *p* = 0.024) and for study participants living in Eastern Europe (OR 1.69, *p* < 0.001, only in the first year of the program). Furthermore, the odds of study participants benefitting based on the reductions observed in their serum insulin were higher for younger participants (<40 years of age) (OR 1.51, *p* = 0.016) and those living in Eastern Europe (OR 1.85, *p* = 0.004). In contrast, study participants from Southern Europe (OR 0.56, *p* = 0.001) had lower odds of benefitting from the Feel4Diabetes program. Lastly, the odds of study participants benefitting based on the reductions observed in their HbA1c were higher in those that received the more intensive intervention (OR 1.90, *p* = 0.002, only in the first year of the program) and those living either in Southern (OR 3.67, *p* < 0.001) or Eastern Europe (OR 2.47, *p* < 0.001, only in the first year of the program).

## 4. Discussion

The present study aimed to identify baseline socio-demographic characteristics and body weight perceptions of study participants that benefitted most from the 2-year Feel4Diabetes program based on favourable changes in their anthropometric and glycaemic profile indices. Overall, study participants from Bulgaria (i.e., LMIC in Eastern Europe) and Spain (i.e., HIC in Southern Europe that was under austerity measures between 2010–2014 due to the economic crisis) were mainly the ones that benefitted the most, either during the first or the second year of the program. Specifically, the highest percentages observed were those of participants from Spain and were relevant to benefits based on the reduction in their WC during the first (35.8%) and the second (41.1%) year of the program. These findings potentially reflect the social and economic diversity between Northern and Southern–Eastern Europe, as well as the discrepancy of primary health care services and systems between these regions. Lower SES is generally associated with a higher prevalence of unhealthy behaviours [12,19,30] and limited access to primary care services, especially regarding the prevention of chronic diseases such as T2D ([11]). Therefore, based on the current findings, it can be concluded that Southern–Eastern Europe and low SES regions with population groups at risk for T2D provide a more fertile ground for initiatives like the Feel4Diabetes program to achieve behavioural and lifestyle changes and the corresponding health benefits stemming from them [31,32]. The regional differences with regards to the benefits stemming from the Feel4Diabetes program may also be attributed to the higher baseline values of BMI and WC in countries like Spain, Greece and Finland, compared to Belgium and Bulgaria, which left space for greater weight loss and consequently additional related benefits for glycaemia. In addition, the observed regional differences may also be due to environmental and genetic differences of the local population groups that were the recipients of the program, the level of acceptance and incorporation of the behavioural change advice in everyday lifestyle, as well as in the way anthropometry and lab analyses were conducted in the different study centres. However, the procedures followed for implementing the program and for collecting data and analysing blood were standardised in all study centres, which used the same protocols and relied on research team members that were centrally trained in order to minimise any inter-observed variability [20].

The present study also showed that those who received the more intensive intervention had higher odds for reducing their WC and HbA1c. The main implication of this observation is that standard care is not enough to lead to health benefits, thus highlighting the need for more intensive counselling as an effective means to reduce cardiometabolic risk related to central obesity and prevent T2D as evidenced by the improvement in HbA1c. The fact that the counselling sessions were tailored to each country’s needs and prioritised EBRBs that were most relevant and appealing to the target population in each country provides an explanation of why the benefit was higher for those that received the more intensive intervention. In this context, some examples of advice delivered to participants via the counselling sessions for increasing physical activity was to walk in the forest in Finland or to visit and exercise in the schoolyard in Greece, since school yards were made accessible after school hours, or to cook based on healthy recipes and use walking routes promoted by NGOs and government agencies in Spain.

Regarding the socio-demographic characteristics of participants who benefitted most, the present study showed that the Feel4Diabetes program was more effective in younger adults, in men, in those who had a tertiary education and who were unemployed. Contrary to our findings, other studies have shown that women and older adults are more likely to participate to nutrition programs [33], mainly due to the greater desire to lose weight in women compared to men and a greater time availability in older compared to younger adults. Men are affected more by T2D worldwide and in fact are diagnosed at a younger age and at lower levels of BMI than women [34]. Nevertheless, most intervention programs targeting the prevention of T2D give only limited information about the influence of gender, although some of them found that females benefitted, less probably because of increasing difficulties in adhering to the proposed lifestyle changes and because psychosocial stress appears to have greater impact on women rather than on men [34,35]. From a physiology perspective, some other studies reported that men show more pronounced improvements in their cardiometabolic risk profile following behavioural change interventions for the prevention of diabetes, because they mobilise more intra-abdominal fat compared to women [36,37]. The higher effectiveness of the Feel4Diabetes program among more educated study participants is probably indicative of the ability of these individuals to better understand and more successfully adopt the messages delivered to them through the counselling sessions, to embed them in their lifestyle and thus achieve favourable behavioural changes [38,39,40,41]. The fact that unemployed participants were also more likely to benefit from the program is probably indicative of time availability that allowed them to attend the counselling session, as well as of lower socio-economic status that provides some space for improvement in unhealthy behaviours and consequently in health indices. In this context, unemployment has been associated with lifestyle risk factors, such as an unhealthy diet, lack of exercise, increased smoking and alcohol abuse [42], and also with other inappropriate health behaviours stemming from changes in social relationships and psychological disorders which follow financial hurdles [43]. The success of the Feel4Diabetes program in benefitting unemployed individuals reflects one of the program’s main objectives, which was to identify those at higher risk for T2D and provide the appropriate support and behavioural change advice that would help them improve risky behaviours and as such prevent the onset of the disease.

Personal attributes, beliefs and perceptions are also known to determine behaviours related to health outcomes [41,44]. In this regard, overweight adults who perceive their body weight as higher than normal, thus acknowledging their excess body weight, are more motivated to take action to improve their weight status [45]. This provides the basis for explaining the higher odds of benefit, based on the reduction of anthropometric indices, in those that perceived their body weight as higher than normal at baseline. 

The findings of the present study should be interpreted taking into consideration its strengths and limitations. First, the Feel4Diabetes program did not follow a ‘one size fits all’ approach; rather, from a preselected list of targeted EBRBs identified from the literature, the participating countries, schools and families could choose and prioritise the most relevant and appealing EBRB for themselves via the SMART goals approach. Furthermore, the behavioural change messages were tailored according to each country’s needs and context, while the large study sample, the relatively long (i.e., 2-year) duration of the program and the standardised protocols and procedures followed across all centres to objectively collect a huge number of data safeguard an accurate and reliable assessment and increase the internal and external validity of the study findings. Regarding limitations, part of the collected data was self-reported and as such subjected to recall bias and social desirability. Moreover, as Feel4Diabetes was an intervention targeting families and using school as an entry point to recruit participants, our results might not be applicable to single adults or families with no children at all or no primary school children. However, in terms of our target population the results stemming from our study can be generalised to the whole population of adults/families with children attending primary school, also considering that the participation rate of families was quite high [20].

## 5. Conclusions

In conclusion, those living in southern and eastern European countries, those who received the more intensive intervention, younger adults, men and those who had a tertiary education and were unemployed at baseline were more likely to benefit from the Feel4Diabetes program by improving their anthropometric and/or glycaemic profile indices. Furthermore, individuals that perceived their body weight as higher than normal were also more likely to benefit from the program. The practical applications of the associations observed between participants’ characteristics and benefit from the intervention are threefold. Firstly, they point to the sociodemographic profile of the individuals that are more likely to show health benefits from the implementation of lifestyle interventions. Furthermore, they suggest higher intervention effectiveness when implemented in southern and eastern European countries which are in greater need for such programs. Finally, they highlight the need for tailoring future interventions to the characteristics, perceptions and beliefs of the target populations in order to increase their effectiveness.

## Figures and Tables

**Table 1:** Descriptive characteristics of the study participants.

	Total Sample (*n* = 2294)	More Intensive Intervention (*n* = 1284)	Standard Care (*n* = 1010)	*p*-Value *
**Socio-demographics**				
Gender	%	%	%	
Males	34.4	35.3	34.0	0.425
Females	65.6	64.7	66.0	
Region	%	%	%	
Northern Europe, HIC	26.8	25.1	28.9 ^†^	0.019
Eastern Europe, LMIC	30.7	32.2	28.8	
Southern Europe, HIC under austerity	42.5	42.7	42.3 ^†^	
Education	%	%	%	
≤12 years of education	26.8	25.9	28.5	0.108
>12 years of education	73.2	74.1	71.5	
Marital status	%	%	%	
Not married	8.3	9.1	7.7	0.157
Married	91.7	90.9	92.3	
Race	%	%	%	
Non-Caucasian	5.8	5.6	6.0	0.709
Caucasian	94.2	94.4	94.0	
Employment status	%	%	%	
Unemployed	21.8	21.1	23.0	0.234
Employed	78.2	78.9	77.0	
	Mean (SD)	Mean (SD)	Mean (SD)	
Age (years)	42.3 (7.6)	42.9 (7.6)	41.5 (7.1)	0.114
**Anthropometrics**	Mean (SD)	Mean (SD)	Mean (SD)	
Body weight (Kg)	81.5 (18.5)	81.3 (18.6)	81.9 (18.6)	0.342
Height (cm)	168.3 (9.3)	168.1 (9.4)	168.5 (9.7)	0.322
BMI (kg/m^2^)	28.7 (5.7)	28.6 (5.4)	28.8 (5.7)	0.550
WC (cm)	94.4(14.7)	95.4 (14.0)	93.8 (15.0)	0.269
**Glycaemic profile indices**	Mean (SD)	Mean (SD)	Mean (SD)	
Fasting plasma glucose (mmol/L)	5.3 (1.1)	5.3 (1.2)	5.3 (1.1)	0.563
Fasting serum insulin (mU/L)	9.3 (8.8)	9.5 (9.5)	9.1 (8.1)	0.332
HBA1c	5.5 (0.6)	5.6 (0.6)	5.4 (0.6)	0.611

BMI: Body Mass Index; WC: Waist Circumference; HBA1c: Glycosylated Haemoglobin A1c. SD: Standard Deviation; HIC: High Income Country; LMIC: Low and Middle Income Country; * *p*-values for testing between-group differences in continuous variables were derived from the independent samples *t*-test. *p*-values for examining associations between categorical variables were derived from the Chi-square test. ^†^ *p* < 0.005 based on the chi-square test for pairwise comparisons between percentages.

**Table 2:** Mean anthropometric values at baseline, mean weight loss and percentages of participants (%) benefitting from the Feel4Diabeets program in the total sample, by country and duration of the program.

	1 Year of the Feel4Diabetes Program					
	Total (*n* = 978)	Finland (*n* = 144)	Belgium (*n* = 126)	Bulgaria (*n* = 199)	Spain (*n* = 206)	Greece (*n* = 288)
BMI ^†^ (Kg/m^2^) at baseline	28.7 (5.7)	29.3 (4.7) ^a^	27.7 (5.0) ^a,b,c^	27.5 (5.8) ^a,b,c^	29.2 (5.3) ^b^	29.3 (5.8) ^c^
WC ^†^ (cm) at baseline	94.4(14.7)	98.6 (12.2) ^a^	91.9 (14.2) ^a,b,c^	90.4 (15.7) ^a,b,c^	98.6 (12.8) ^b^	95.9 (14.3) ^c^
Weight loss ^†^ (kg) in those benefitted	7.9 (4.5)	8.1 (3.5)	7.5 (3.8)	7.6 (3.7)	8.6 (6.1)	7.7 (3.9)
% of participants benefitted * based on:						
Body weight (%)	11.2	6.9	9.5	16.6	14.6	8.0
WC (%)	17.2	16.7	18.3	6.6	35.8	11.8
Fasting plasma glucose (%)	21.3	18.9	22.0	25.8	23.4	16.5
Serum Insulin (%)	15.0	7.0	18.4	23.9	17.4	9.1
HBA1c (%)	19.2	2.4	N/A	25.9	20.8	N/A
	**2 Years of the Feel4Diabetes Program**					
	**Total** **(*n* = 755)**	**Finland** **(*n* = 123)**	**Belgium** **(*n* = 89)**	**Bulgaria** **(*n* = 126)**	**Spain** **(*n* = 195)**	**Greece** **(*n* = 230)**
Weight loss (kg) in those benefitted	8.9 (6.7) ^†^	8.9 (6.7) ^†^	9.0 (5.5) ^†^	7.7 (3.5) ^†^	10.7 (9.6) ^†^	8.5 (5.9) ^†^
% of participants benefitted * based on:						
Body weight (%)	15.2	13.0	18.8	22.2	12.4	13.9
WC (%)	24.0	23.6	14.5	4.8	41.1	24.3
Glucose (%)	19.6	15.5	20.9	12.1	25.0	19.3
Insulin (%)	12.2	9.7	19.1	16.1	6.7	13.2
HBA1c (%)	13.7	1.0	N/A	8.9	22.1	N/A

WC: Waist Circumference; HBA1c: Glycosylated Haemoglobin A1c. N/A: Not available for this country. * Benefit was defined as a ≥5% reduction in any of the biomarkers summarized in Table 1. † Data presented as mean (sd). Mean values sharing the same superscript letter (i.e., ^a^, ^b^ or ^c^) are statistically significantly different between them (*p*-value < 0.05, derived from one-way ANOVA).

**Table 3:** Odds of achieving a 5% decrease in anthropometric values during the 2 years of the Feel4Diabetes program by participants’ characteristics.

	Odds of Achieving 5% Decrease in:					
	Body Weight			WC		
Characteristics of Study Participants	OR	95% CI	*p*-Value	OR	95% CI	*p*-Value
Treatment (more intensive intervention)	1.25	0.92, 1.69	0.153	**1.53**	**1.17, 1.99**	**0.002**
Age (<40 years)	**1.49**	**1.10, 2.03**	**0.011**	1.09	0.84, 1.45	0.511
Gender (Male)	1.03	0.74, 1.44	0.845	0.85	0.64, 1.14	0.275
Region (Southern HIC under austerity)	1.12	0.82, 1.53	0.480	**2.39**	**1.79, 3.18**	**<0.001**
Region (Eastern LMIC)	**1.56 ^†^**	**1.09, 2.24**	**0.016**	**0.13**	**0.07, 0.26**	**<0.001**
Marital status (Married)	0.81	0.46, 1.41	0.450	0.89	0.55, 1.45	0.640
Race (Caucasian)	1.25	0.57, 2.77	0.579	0.71	0.40, 1.28	0.255
Education (>12 years)	0.84	0.58, 1.22	0.369	**2.06**	**1.40, 3.05**	**<0.001**
Employment status (Unemployed)	**1.62 ^†^**	**1.10, 2.39**	**0.014**	1.04	0.73, 1.49	0.817
Weight perception (Higher than normal)	**3.00**	**1.83, 4.91**	**<0.001**	**1.58**	**1.12, 2.23**	**0.010**

Multivariate logistic regression models were used to examine the associations of study participants characteristics at baseline in those that benefitted from the Feel4Diabetes program. Adjustments were made for age, gender, country and treatment arm (except when used as the dependent variable in the analyses. All odds ratios provide associations for benefits observed in the 2 years of the Feel4Diabetes program, unless if otherwise indicated by the superscript symbol (^†^). This symbol indicates that the odds ratio refers only to the first year of the program. Numbers presented in bold font highlight the statistically significant odds ratios.

**Table 4:** Odds of achieving a 5% decrease in glycaemic profile values during the 2 years of the Feel4Diabetes program by participants’ characteristics.

	Odds of Achieving 5% Decrease in:								
	Fating Plasma Glucose			Fasting Serum Insulin			HbA1c		
Characteristics of Study Participants	OR	95% CI	*p*-Value	OR	95% CI	*p*-Value	OR	95% CI	*p*-Value
Treatment (more intensive intervention)	1.16	0.88, 1.53	0.292	1.11	0.80, 1.54	0.541	**1.90 ^†^**	**1.28, 2.82**	**0.002**
Age (<40 years)	0.85	0.64, 1.13	0.255	**1.51**	**1.08, 2.10**	**0.016**	1.10	0.68, 1.76	0.702
Gender (Male)	**1.40**	**1.05, 1.86**	**0.024**	0.96	0.66, 1.38	0.808	1.31	0.80, 2.13	0.283
Region (Southern HIC under austerity)	1.20	0.90, 1.59	0.219	**0.56**	**0.40, 0.089**	**0.001**	**3.67**	**2.25, 5.99**	**<0.001**
Region (Eastern LMIC)	**1.69 ^†^**	**1.28, 2.23**	**<0.001**	**1.85**	**1.21, 2.81**	**0.004**	**2.47 ^†^**	**1.70, 3.58**	**<0.001**
Marital status (Married)	0.95	0.56, 1.60	0.833	1.32	0.64, 2.84	0.451	0.81	0.38, 1.71	0.575
Race (Caucasian)	0.89	0.48, 1.66	0.710	2.97	0.70,12.56	0.139	1.35	0.62, 2.95	0.446
Education (>12 years)	0.97	0.70, 1.36	0.849	0.95	0.63, 1.42	0.784	1.12	0.51, 2.47	0.782
Employment status (Unemployed)	0.93	0.63, 1.37	0.718	1.19	0.77, 1.84	0.429	1.37	0.75, 2.53	0.308
Weight perception (>40 years)	0.94	0.68, 1.30	0.687	0.13	0.51, 1.09	0.132	1.18	0.69, 2.03	0.551

Multivariate logistic regression models were used to examine the associations of study participants characteristics at baseline in those that benefitted from the Feel4Diabetes program. Adjustments were made for age, gender, country and treatment arm (except when used as the dependent variable in the analyses). All odds ratios provide associations for benefits observed in the 2 years of the Feel4Diabetes program, unless if otherwise indicated by the superscript symbol (^†^). This symbol indicates that the odds ratio refers only to the first year of the program. Numbers presented in bold font highlight the statistically significant odds ratios.
